# COVID-19 infection control protocol and acceptance in an ART in vitro fertilization hospital

**DOI:** 10.1186/s43043-022-00112-8

**Published:** 2022-07-14

**Authors:** Yu Horibe, Akira Nakabayashi, Shuko Murata, Tomomi Hashimoto, Tsutomu Tabata

**Affiliations:** Department of Obstetrics and Gynecology, Tokyo Women’s Medical University, Tokyo, Japan

**Keywords:** Assisted reproductive technology, Embryo transfer, Oocyte retrieval, Polymerase chain reaction, Severe acute respiratory syndrome coronavirus 2

## Abstract

**Purpose:**

In Tokyo, where the highest coronavirus disease 2019 (COVID-19) infection rates have been reported nationally, we introduced and performed polymerase chain reaction (PCR) testing on the patients prior to them coming for oocyte retrieval (OR) or embryo transfer (ET) procedures. In addition, we recommended that patients self-inject ovarian stimulation drugs to reduce the number of hospital visits required. We aimed to assess the patient acceptance of these measures and the change of treatment number.

**Methods:**

We conducted a retrospective study examining the patients coming for OR or ET, from the first time a state of emergency was declared in Japan, May 2020, until September 2021.

**Results:**

A total of 79 out of 94 (94%) patients complied with the measures. This may reflect that PCR universal screening was accepted by most patients as necessary for reducing infection spread. In addition, the number of patients receiving OR and ET increased. The widespread adoption of work-from-home practices during the pandemic has made outpatient visits more acceptable to the general public.

**Conclusions:**

Universal screening and self-injection are accepted and effective infection measures in patients presenting for OR and ET.

## Introduction

During the coronavirus disease 2019 (COVID-19) pandemic, medical staff engaged in assisted reproductive technology (ART) treatment faced problems with administering medical treatment and respecting infection control. In ART hospitals globally, several protocols have been implemented, including restricting patient numbers in waiting rooms, removal of semen collection rooms, and online counseling. However, each facility has devised its own set of protocols [1].

In Japan, the first significant spread of COVID-19 started in March 2020, with the first state of emergency being declared on 7 May 2020. At our hospital in Tokyo, patients who were possibly infected with COVID-19 were prohibited from entering outpatient wards through temperature checking and vetting by a written questionnaire before entry.

For patients who must be admitted for inpatient treatment, such as surgery, oocyte retrieval (OR), or egg transfer, a polymerase chain reaction (PCR) test with a negative result no more than 7 days before undergoing the procedure is required. Moreover, we recommended and encouraged the use of self-injection of drug treatments to reduce the number of hospital visits required.

Herein, we evaluate patient acceptability by clarifying the change of treatment number towards our COVID-19 screening protocol.

## Materials and method

We conducted a retrospective study in a single hospital, covering 202 patients who received ART treatment at Tokyo Women’s Medical University Hospital from May 2020 to September 2021. We considered the patient age and background. We then calculated the number of positive COVID-19 PCR results and their rate (100% of the patients were asked to and agreed to take the PCR test), rate of self-injection, and changes in the number of treatment visits (generally, a PCR test was not required before admission at other hospitals). The value of self-injection was personalized by the patient’s condition. The method of the PCR test and protection was referred to the American Society for Reproductive Medicine (ASRM) [2]. There are no exclusion criteria in this study.

## Results

**Fig. 1:**
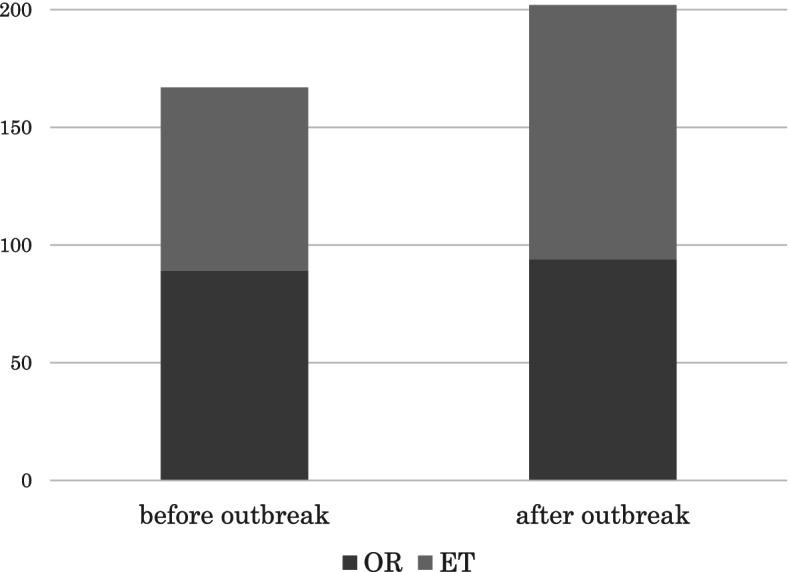
A cChange of oocyte retrieval and embryo transfer cycles before and after the pandemic. A cChange of oocyte retrieval (OR) and embryo transfer (ET) cycles before and after the outbreak. Both the number of OR and ET increased, and the number of treatments in patients aged over 40 remarkably increased after the outbreak. Polymerase chain reaction testing was implemented for all patients. The rRate of self-injection was 84%

The average recorded age of patients was 39.8 ± 3.8 years, with 94 OR cycles and 108 embryo transfers (ETs) being performed out of a total of 202 treatment cycles (Fig. 1). Table 1 shows a breakdown of the numbers and types of controlled ovarian stimulation (COS) treatments performed and the number of each diagnosed cause of infertility, which show a wide variety. The mean number of eggs acquired, the number of eggs fertilized per OR, and the number of pregnancies achieved for patients under the age of 40 are also displayed. On some occasions, due to the status of the pandemic and other factors, the ART scheduling was interrupted. Of the 202 patients who underwent PCR testing, one patient tested positive for COVID-19. For administration of injections, 79 cycles of self-injection were performed out of a total of 94, with 13 cycles of CC-gonadotropin and 2 cycles of gonadotropin ultimately being administered by the medical staff. The number of confirmed treatment cycles increased compared to that of the previous COVID-19 pandemic during the same period: the number of ORs was 62 cycles and that of ET was 58 cycles. Procedures performed in patients over 40 years of age increased from 47 to 112, while in those under 40 years of age, increased from 73 to 90 procedures.

**Table 1:** Characteristics of study shows number of controlled ovarian stimulation, number of cause of infertility, mean number of eggs acquired, mean number of fertilizations, and pregnancy rate

Age (years)	39.8 ± 3.8
Controlled ovarian stimulation	Cycles
Clomiphene citrate	7
CC + gonadotropin	39
Flare protocol	15
Antagonist method	13
Gonadotropin	5
Progestin-primed ovarian stimulation	25
Cause of infertility	
Unexplained	144
Fallopian tube	14
Endometriosis	4
Male factor	19
Premature menopause	1
Medical justification	3
Mean number of eggs acquired	4.9 ± 4.8
Mean number of fertilizations	3.1 ± 3.7
Pregnancy rate	23.1% (25/108)
Pregnancy rate under the age of 40 years	27.0% (17/63)

## Discussion

The medical practice has changed dramatically due to the COVID-19 pandemic. The psychological burden on the patients has become a significant issue. One study conducted in England showed that the postponement of ART treatment adversely affected patients’ stress levels [3]. Enhancing communication between patients and staff had the opposite effect. In Italy, a study showed patients undergoing ART treatment during the “lock down” period experienced more issues with psychological deterioration, body weight, and heart disease [4]. Another study showed that, as the pandemic was brought under control, the mental health of women undergoing ART treatment improved. This may indicate that the best course of action is to continue the treatment whilst making an appropriate adjustment to practices to overcome constraints introduced by the COVID-19 pandemic [5].

During the pandemic, one ART Hospital in New York performed screening on all 1700 of its patients, with 7 patients testing positive for the virus. A low positivity rate serves to show that ART treatment under the pandemic is sufficiently viable [6]. In practice, the probability of severe COVID-19 infection amongst women of reproductive age is lower than that of males as well as women of other age groups [7]. Continuing with universal screening is necessary because of the COVID-19 mutability and possible resurgence. Moreover, if a hospital suffers a cluster infection, all patient treatments would need to be halted [8].

Reasons for 100% compliance and negative result rate of PCR tests performed may include our hospital location, Shinjuku district, which was the area affected most severely in terms of infection concentration; hence, PCR testing was made mandatory for all patients as a standard hospital policy. It was therefore relatively easy to gain patient acceptance of the policy, as it was done already. As a general characteristic, many university hospital patients suffer from chronic diseases and are at a higher risk for the COVID-19 infection. Despite this, the number of treatments being performed before and after the pandemic surge and the implementation of safety measures remained roughly the same. This may indicate patients’ desire not to have their treatment delayed, while accepting that PCR testing minimizes their risk of COVID-19 infection.

The limitation of the study is that this is only a single-institution study, and universal screening, PCR testing, and IVF protocol depend on each institution.

Another factor may be that changes in lifestyle due to the pandemic, like remote working, have improved people’s acceptance of and their access to outpatient treatment, which, in turn, increased the demand of in vitro fertilization cycles [9].

Other institutions have also adopted protective measures; for example, abolition of sperm collection at ART hospitals in favor of the collection from home, performing online consultations, and keeping a distance of 6 ft. between patients in waiting rooms have all been implemented in various institutions. Such measures have yielded success in infection control comparable to those achieved as a result of our policies. Conversely, some others have experienced difficulty in achieving reasonable protection with their protocols, such as advocating avoidance of travel by public transportation, changing medical staff hours to avoid peak travel times, and prohibiting hospital visits except for the patients requiring ART treatment [10].

At our institution, we recommended self-injection of a recombinant follicle-stimulating hormone to reduce the number of hospital visits the patient had to make, thus reducing the infection risk. Elsewhere, Carlo (see Reference [1, 11]) proposed a treatment guideline for a group of 2–4 patients with low prognosis in the POSEIDON group (i.e., those who exhibited poor ovarian response due to age, antimüllerian hormone, or FC), with the objective that their fertility treatment must not be delayed. The risk of COVID-19 transmission to staff and others can be minimized through the use of personal protective equipment protocols [1, 11].

Meng reported there was a small drop in blastocyst achievement in patients positive for COVID-19; however, there were no changes to chemical pregnancy, clinical pregnancy, implantation, or early abortion rates amongst COVID-19-positive patients with mild to moderate symptoms [12]. Conversely, reports showed that 25% of males recovering from COVID-19 infection suffer azoospermia or oligospermia; hence, it is still important to control the spread of the infection, even speaking from a strict fertility point of view [13].

Recently, it has become unclear whether the spread of COVID-19 can be contained through vaccination alone [14]. Nonetheless, the American Society for Reproductive Medicine (ASRM) recommends a booster shot be taken during early-stage pregnancy since it has been shown to have no impact on fetus defect rates [2]. So far, Japan has experienced six waves of COVID-19 infection. We, therefore, recommend all ART patients be administered the vaccine and proactive use of self-injection, whilst continuing to use universal screening and self-injection to further reduce the risk of infection.

## Data Availability

Data sharing is not applicable to this article as no datasets were generated or analyzed during the current study.

## Abbreviations

PCR: Polymerase chain reaction
OR: Oocyte retrieval
ET: Embryo transfer
ART: Assisted reproductive technology
ASRM: American Society for Reproductive Medicine
COS: Controlled ovarian stimulation
